# Inhibition of circulating exosomal microRNA-15a-3p accelerates diabetic wound repair

**DOI:** 10.18632/aging.103143

**Published:** 2020-05-21

**Authors:** Yuan Xiong, Lang Chen, Tao Yu, Chenchen Yan, Wu Zhou, Faqi Cao, Xiaomeng You, Yingqi Zhang, Yun Sun, Jing Liu, Hang Xue, Yiqiang Hu, Dong Chen, Bobin Mi, Guohui Liu

**Affiliations:** 1 Department of Orthopedics, Union Hospital, Tongji Medical College, Huazhong University of Science and Technology, Wuhan 430022, China; 2 Department of Orthopedic Surgery, Tongji Hospital, Tongji University School of Medicine, Shanghai 200065, China; 3 Department of Orthopedic Surgery, Brigham and Women’s Hospital, Harvard Medical School, Boston, MA 02125, USA; 4 Department of Neurosurgery, Union Hospital, Tongji Medical College, Huazhong University of Science and Technology, Wuhan 430022, China

**Keywords:** microRNA-15a-3p, exosome, diabetic foot ulcer, wound repair, NADPH oxidase 5

## Abstract

Diabetic foot ulcers are a common complication of diabetes, and are usually incurable in the clinic. Exosomes (carriers that transfer endogenous molecules) from diabetic patients’ blood have been demonstrated to suppress diabetic wound repair. In this study, we investigated the effects of circulating exosomal microRNA-15a-3p (miR-15a-3p) on diabetic wound repair. Exosomes were extracted from diabetic patients’ blood, and were found to inhibit diabetic wound repair *in vitro* and *in vivo*. miR-15a-3p was upregulated in diabetic exosomes, and impaired wound healing. When miR-15a-3p was knocked down in diabetic exosomes, their negative effects were partially reversed both *in vitro* and *in vivo*. NADPH oxidase 5 (*NOX5*) was identified as a potential target of miR-15a-3p, and the inhibition of *NOX5* reduced the release of reactive oxygen species, thereby impairing the functionality of human umbilical vein endothelial cells. In summary, inhibition of circulating exosomal miR-15a-3p accelerated diabetic wound repair by activating *NOX5*, providing a novel therapeutic target for diabetic foot ulcer therapy.

## INTRODUCTION

As the dietary structure changes and mental and social pressures increase, the prevalence of diabetes continues to rise [1]. Patients who have had diabetes for longer than three years are more susceptible to chronic complications [2]. Diabetic foot ulcer (DFU) is one of the most serious chronic complications of diabetes, and accounts for about 4% of diabetic complications [3]. Although many therapeutic strategies have been developed to treat DFU, the clinical outcomes of DFU are still unsatisfactory [4, 5].

Exosomes – extracellular vesicles with diameters of 30-150 nm – can be derived from various cell types, and potently regulate numerous processes *in vivo* [5, 6]. For example, lymphocyte-derived exosomes contribute to the development of type 1 diabetes by promoting pancreatic β-cell death [7]. In addition, circulating diabetic exosomes are associated with the delayed healing of diabetic wounds [8]. MicroRNAs (miRNAs) are small non-coding RNA molecules that can activate or inhibit a variety of biological processes [9]. MiRNAs can be secreted from exosomes and transferred to proximal or distal target cells to downregulate gene expression [10]. Exosomal miR-15a derived from the pancreas has been reported to aggravate diabetic complications by inducing oxidative stress [11].

Isoforms of the NADPH oxidase (NOX) family participate in microbial killing, neutrophil chemotaxis and signal transduction for essential processes of cutaneous wound healing [12, 13]. Oxygen is vital for each stage of wound healing because such activities rely on energy from adenosine triphosphate, which is generated through oxidative phosphorylation. However, oxygen can also be reduced to superoxide and initiate the formation of other reactive oxygen species (ROS), which subsequently react with inorganic molecules, proteins and nucleic acids [14]. Previous studies have indicated that the activation of NOX and the release of ROS are associated with cutaneous wound repair [15, 16].

In this study, we determined the relationship between circulating exosomal miR-15a-3p and *NOX5*, and assessed their effects on diabetic wound repair.

## RESULTS

### Characteristics of exosomes derived from non-diabetic and diabetic patients

**Figure 1:**
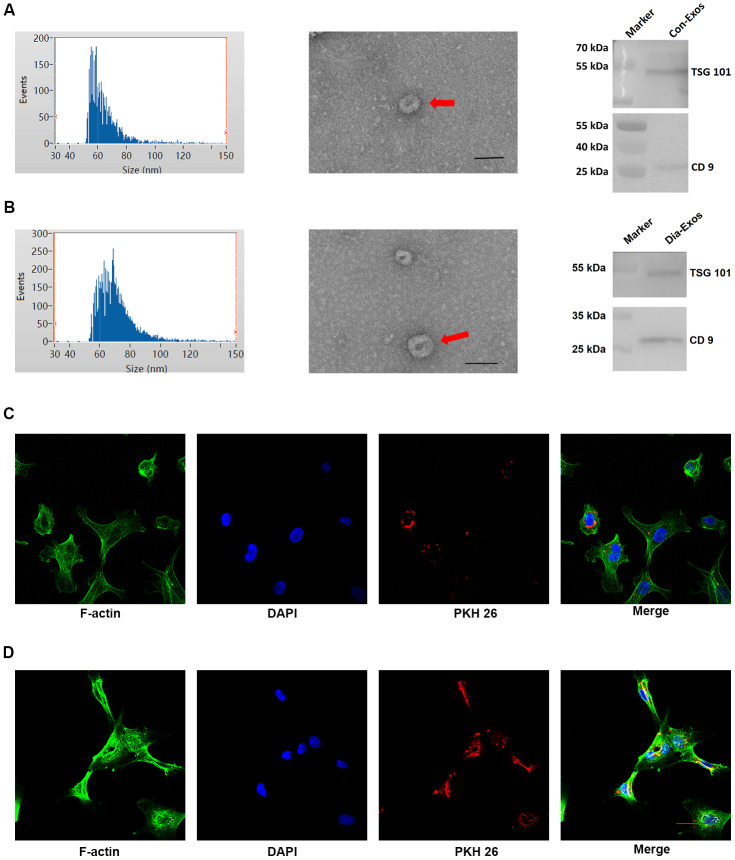
**Features of exosomes derived from non-diabetic and diabetic patients.** (**A**) DLS results, TEM images and WB results of Con-Exos. (**B**) DLS results, TEM images and WB results of Dia-Exos; scale bar: 100 nm. (**C**) PKH26-labeled Con-Exos were absorbed by HUVECs, as indicated by a red fluorescent signal. (**D**) PKH26-labeled Dia-Exos were taken up by HUVECs, as indicated by a red fluorescent signal.

We obtained exosomes from the peripheral blood of non-diabetic foot wound patients (Con-Exos) and DFU patients (Dia-Exos), and examined their features through dynamic light scattering (DLS), transmission electron microscopy (TEM) and Western blotting (WB). The DLS images revealed that the Con-Exos ranged from 30 to 150 nm in size. TEM indicated that these particles had a cup- or sphere-shaped morphology. WB analysis indicated that the Con-Exos expressed exosomal surface markers such as CD9 and TSG101 (Figure 1A). The Dia-Exos exhibited similar features in DLS, TEM and WB analyses (Figure 1B).

We next assessed the ability of human umbilical vein endothelial cells (HUVECs) to take up the circulating exosomes *in vitro.* Con-Exos or Dia-Exos were labeled with PKH26 dye and added to the HUVEC culture medium. The results suggested that HUVECs could take up both Con-Exos and Dia-Exos (Figure 1C and 1D). Thus, the nanosized particles isolated from blood were exosomes that could be taken up by HUVECs.

### Dia-Exos delayed cutaneous wound healing *in vivo*

**Figure 2:**
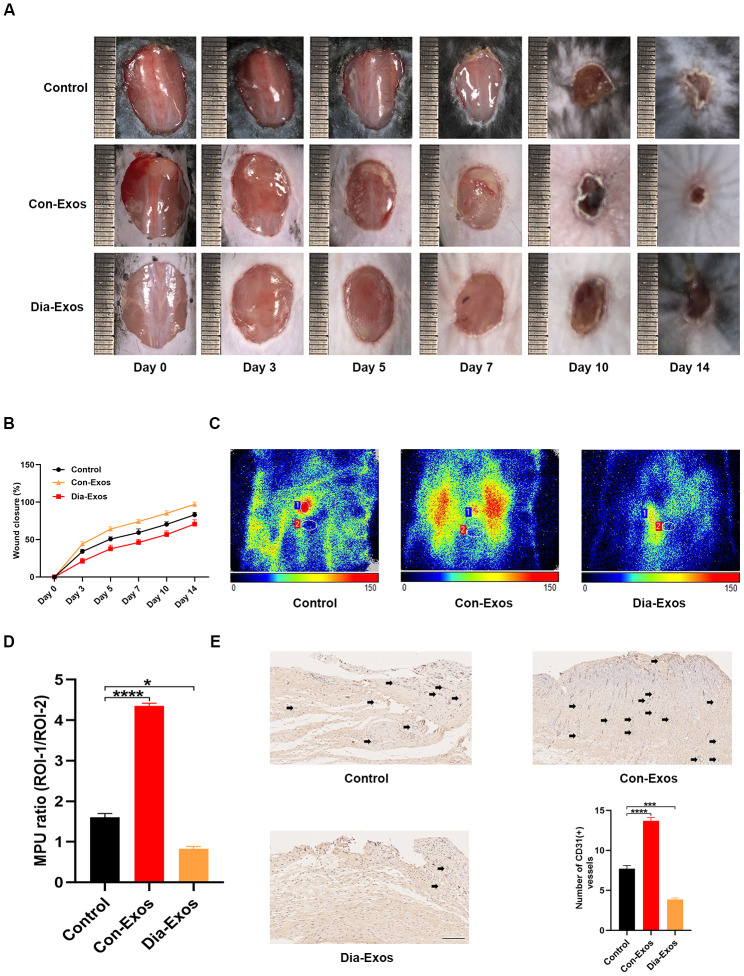
**Dia-Exos hindered wound healing *in vivo.*** (**A**) General view of wound closure after different treatments. Wounds are shown on days 0, 3, 5, 7, 10 and 14 post-wounding. (**B**) The wound closure rates of the three groups; n = 6 per group. (**C**, **D**) The MPU ratio at the wound area in each group was assessed through small animal doppler detection. The MPU ratio is the MPU of the wound area (region of interest 1) divided by the MPU of the area around the wound (region of interest 2). n = 6 per group. (**E**) Immunohistochemical analysis of CD31 in the wound site after different treatments. The number of CD31-positive cells was quantified in five random fields of view. Vessels with diameters of 2-10 μm were counted as individual vessels. n = 6 per group; scale bar: 100 μm. Data are the means ± SDs of three independent experiments. *p < 0.05, **p < 0.01, ***p < 0.001.

Next, we evaluated the influence of Dia-Exos on wound repair by generating full-thickness cutaneous wounds on the backs of mice and locally injecting them with equal volumes of either phosphate-buffered saline (PBS), Con-Exos or Dia-Exos. The general appearance of the wounds indicated that the wound closure rate was significantly slower in the Dia-Exos group than in the Con-Exos group (Figure 2A and 2B). To assess the blood perfusion of the injured area, we performed small animal doppler detection. The results at 10 days post-treatment demonstrated that the mean perfusion units (MPU) ratio at the wound site was lower in the Dia-Exos group than in the Con-Exos group (Figure 2C and 2D). Immunohistochemistry was performed to assess CD31 expression as a marker of newly generated vessels at the wound site. Dia-Exos were found to impair angiogenesis (Figure 2E). These results indicated that Dia-Exos suppressed diabetic wound repair.

### Dia-Exos impaired HUVEC functionality *in vitro*

**Figure 3:**
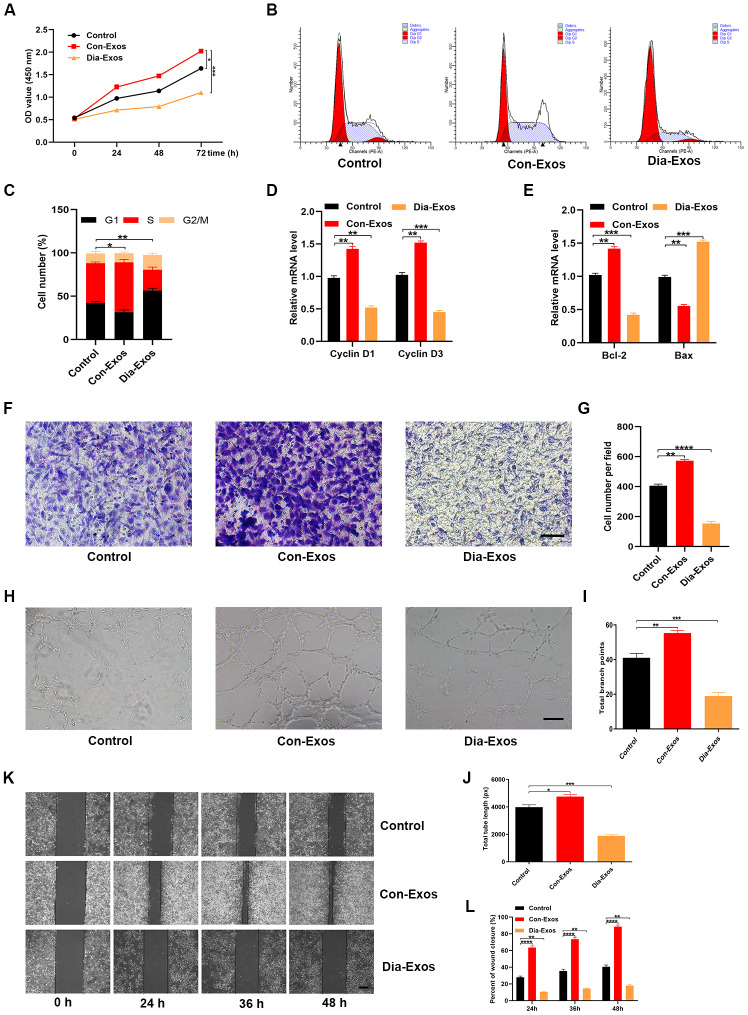
**Dia-Exos impaired HUVEC angiogenesis and survival *in vitro*.** (**A**) The effects of Dia-Exos on HUVEC proliferation were measured with a CCK-8 assay. (**B**, **C**) Flow cytometry was used to quantify the cell cycle distribution. (**D**) The effects of Dia-Exos on the proliferation-related genes *Cyclin D1* and *Cyclin D3* were assessed using qRT-PCR. (**E**) The effects of Dia-Exos on the apoptosis-related genes *Bcl-2* and *Bax* were assessed using qRT-PCR. (**F**, **G**) A Transwell migration assay was used to assess the effects of Dia-Exos on HUVEC migration; scale bar: 100 μm. (**H**–**J**) A tube formation assay was used to assess the effects of Dia-Exos on HUVEC angiogenesis; scale bar: 200 μm. (**K**, **L**) The scratch assay results of the three groups; scale bar: 250 μm. Data are the means ± SDs of three independent experiments. *p < 0.05, **p < 0.01, ***p < 0.001.

We then assessed the effects of Dia-Exos on HUVEC functionality. First, we used a Cell Counting Kit-8 (CCK-8) assay to assess cell proliferation. HUVECs treated with Dia-Exos exhibited a significantly lower extent of proliferation than those treated with Con-Exos (Figure 3A). Flow cytometry was then used to quantify the cell cycle distribution, and the proportion of cells entering S phase was found to be significantly reduced following Dia-Exos treatment (Figure 3B and 3C). The proliferation-related genes *Cyclin D1* and *Cyclin D3* were also downregulated in HUVECs treated with Dia-Exos (Figure 3D). Consistently, *Bcl-2* expression was reduced and *Bax* expression was elevated following Dia-Exos treatment (Figure 3E).

A Transwell migration assay was then performed to test the migration abilities of HUVECs that had received different treatments. The migration ability was weaker in HUVECs treated with Dia-Exos than in those treated with Con-Exos (Figure 3F and 3G). Next, a tube formation assay was performed to assess the effects of the exosomes on HUVEC angiogenesis. Tube formation was significantly reduced in HUVECs treated with Dia-Exos (Figure 3H–3J). In addition, a wound scratch assay was performed to test the effects of the exosomes on wound healing *in vitro*. Dia-Exos significantly impaired cell migration, consistent with the Transwell migration assay results (Figure 3K and 3L). All these findings indicated that Dia-Exos suppressed wound healing *in vitro*.

### MiR-15a-3p was enriched in Dia-Exos and impaired HUVEC functionality

**Figure 4:**
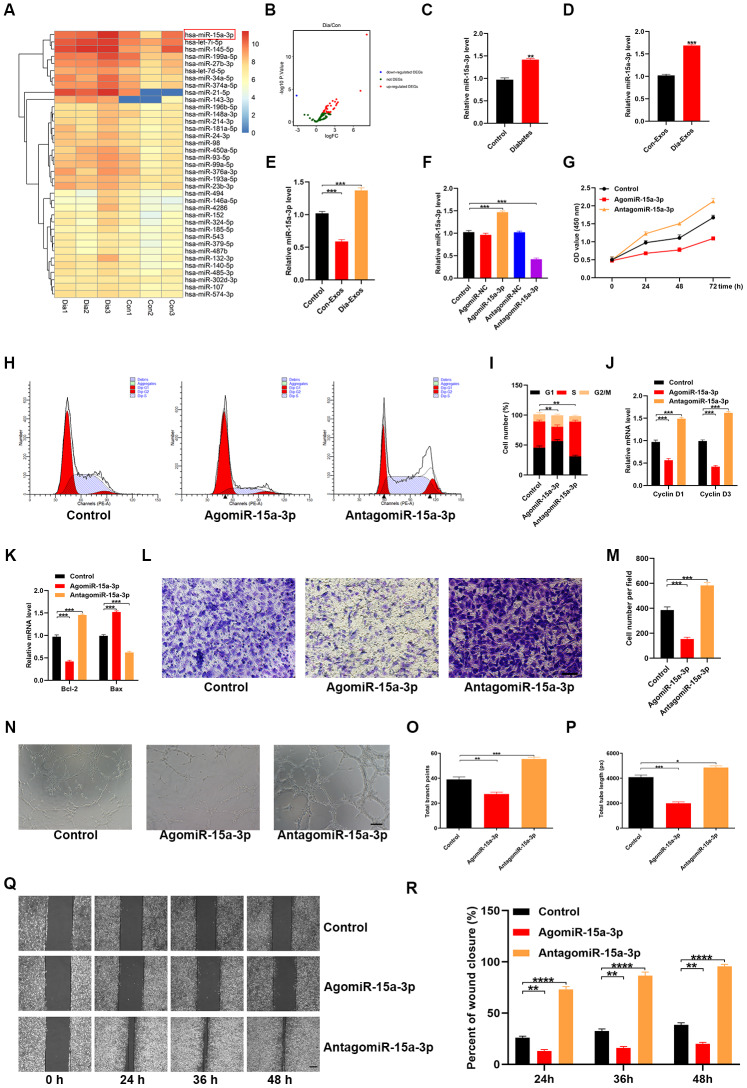
**Dia-Exos were enriched with miR-15a-3p, which altered HUVEC function.** (**A**, **B**) An miRNA microarray dataset of non-diabetic foot wound patients and DFU patients retrieved from NCBI GEO (accession number: GSE80178) indicated that miR-15a-3p was upregulated in foot skin from diabetic patients. (**C**, **D**) MiR-15a-3p overexpression was found in serum and exosomes from the diabetic group; n = 10 per group. (**E**) Effects of the two kinds of exosomes on miR-15a-3p levels in the skin tissues of mice treated with Dia-Exos. (**F**) qRT-PCR indicated that antagomiR-15a-3p could partially counteract the overexpression of miR-15a-3p in HUVECs. (**G**) A CCK-8 assay was used to assess the effects of antagomiR-15a-3p on HUVEC proliferation. (**H**, **I**) Flow cytometry was used to quantify the cell cycle distribution. (**J**) qRT-PCR analysis indicated that antagomiR-15a-3p could restore the mRNA levels of *Cyclin D1* and *Cyclin D3*. (**K**) The effects of antagomiR-15a-3p on the apoptosis-related genes *Bcl-2* and *Bax* were measured using qRT-PCR. (**L**, **M**) A Transwell migration assay was used to measure the effects of miR-15a-3p on HUVEC migration; scale bar: 100 μm. (**N**–**P**) A tube formation assay was used to assess the effects of miR-15a-3p on HUVEC angiogenesis; scale bar: 200 μm. (**Q**, **R**) The scratch assay results of the three groups; scale bar: 250 μm. Data are the means ± SDs of three independent experiments. *p < 0.05, **p < 0.01, ***p < 0.001.

A recent study indicated that exosomal miR-15a-3p expression was elevated in patients with diabetes [11]. We retrieved an miRNA microarray dataset of foot skin samples from non-diabetic foot wound patients and DFU patients from the Gene Expression Omnibus (GEO) of the National Center for Biotechnology Information (NCBI; accession number: GSE80178), which also indicated that miR-15a-3p was upregulated in diabetic patients (Figure 4A and 4B). Thus, miR-15a-3p is a potential target miRNA associated with diabetic wound healing.

Next, quantitative reverse-transcription polymerase chain reaction (qRT-PCR) analyses were performed to determine the expression of miR-15a-3p in both serum samples and exosomes from non-diabetic and diabetic patients. As expected, miR-15a-3p was overexpressed in diabetic serum and exosomes (Figure 4C and 4D). Consistently, miR-15a-3p expression was elevated in the skin tissues of mice treated with Dia-Exos (Figure 4E).

To assess the effects of miR-15a-3p on the functionality of HUVECs, we transfected the cells with an miR-15a-3p agonist or antagonist. The expression of miR-15a-3p was significantly elevated in the agonist group and significantly reduced in the antagonist group (Figure 4F, and Supplementary Figure 1). A CCK-8 cell proliferation assay indicated that the miR-15a-3p agonist significantly reduced HUVEC proliferation (Figure 4G). Flow cytometry was performed to assess the cell cycle distribution, and the results demonstrated that the proportion of cells entering S phase was significantly reduced following miR-15a-3p agonist treatment (Figure 4H and 4I). The proliferation-related genes *Cyclin D1* and *Cyclin D3* were downregulated in HUVECs treated with the miR-15a-3p agonist (Figure 4J). Consistently, *Bcl-2* expression was reduced and *Bax* expression was elevated following miR-15a-3p agonist treatment (Figure 4K).

A Transwell migration assay revealed that the migration ability of HUVECs was reduced after the cells had been treated with the miR-15a-3p agonist (Figure 4L and 4M). HUVEC angiogenesis was assessed through a tube formation assay, which demonstrated that tube formation was significantly reduced in HUVECs treated with the miR-15a-3p agonist (Figure 4N–4P). A wound scratch assay was performed to determine whether miR-15a-3p altered wound healing *in vitro*. We found that the miR-15a-3p agonist significantly suppressed cell migration, consistent with the Transwell migration assay results (Figure 4Q and 4R). These results suggested that miR-15a-3p from Dia-Exos impaired HUVEC functionality.

### Knocking down miR-15a-3p in Dia-Exos partly reversed their inhibition of wound repair

**Figure 5:**
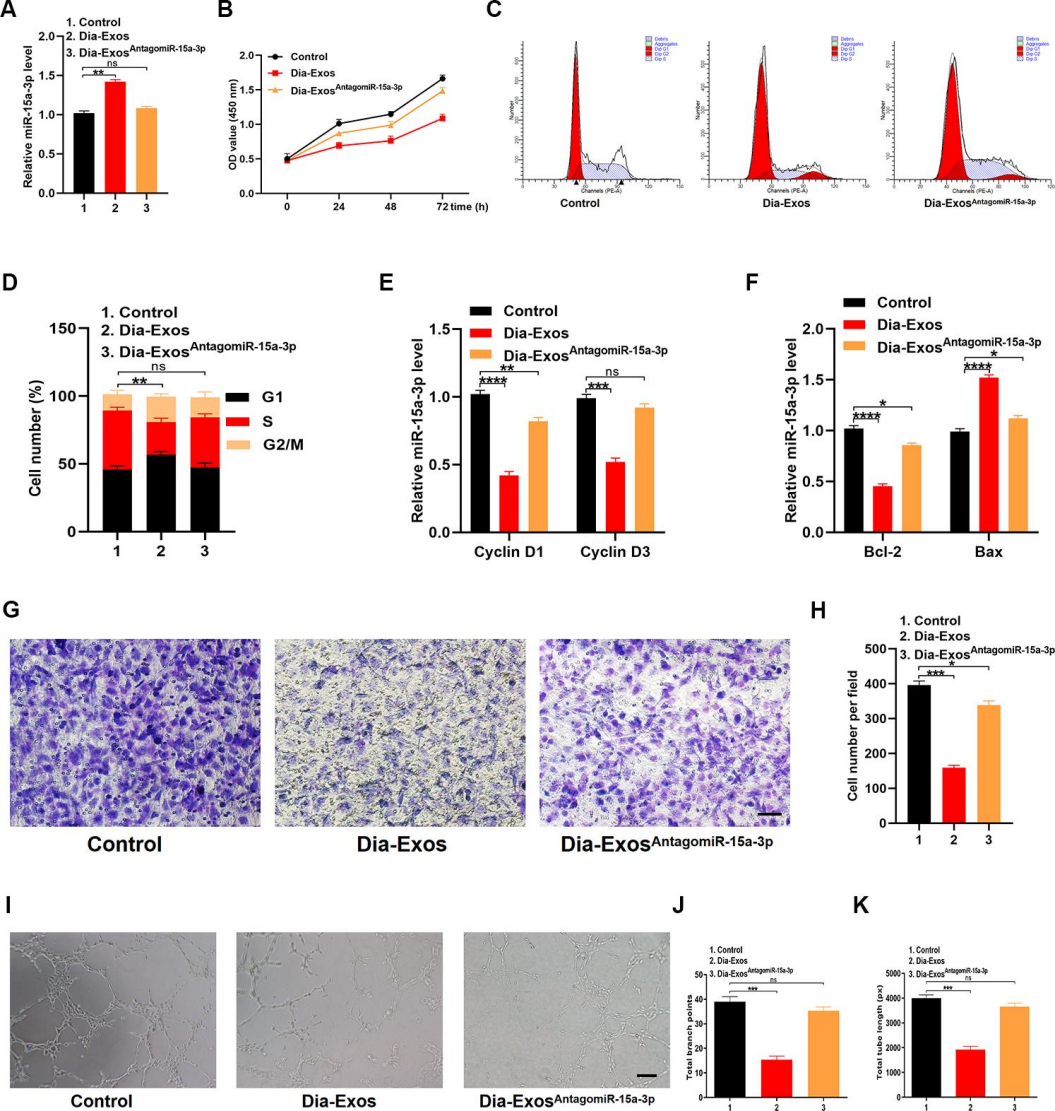
**Inhibition of miR-15a-3p partially reversed the impaired functionality of HUVECs treated with Dia-Exos.** (**A**) MiR-15a-3p levels in the three groups were measured using qRT-PCR. (B) CCK-8 assay results of the three groups. (**C**, **D**) Flow cytometry was used to quantify the cell cycle distribution in treated cells. (**E**) The qRT-PCR results of the proliferation-related genes *Cyclin D1* and *Cyclin D3*. (**F**) The apoptosis-related genes *Bcl-2* and *Bax* were assessed using qRT-PCR. (**G**, **H**) A Transwell migration assay was used to assess the effects of miR-15a-3p inhibition on HUVEC migration; scale bar: 100 μm. (**I**–**K**) A tube formation assay was used to assess the effects of miR-15a-3p inhibition on HUVEC angiogenesis; scale bar: 200 μm. Data are the means ± SDs of three independent experiments. *p < 0.05, **p < 0.01, ***p < 0.001.

HUVECs were randomly divided into three groups: the PBS group (Control), the Dia-Exos group and the Dia-Exos^AntagomiR-15a-3p^ group (Dia-Exos combined with the miR-15a-3p antagonist). We found that miR-15a-3p levels were significantly lower in the Dia-Exos^AntagomiR-15a-3p^ group than in the other groups (Figure 5A). A CCK-8 cell proliferation assay indicated that knocking down miR-15a-3p partially restored the proliferation of HUVECs treated with Dia-Exos (Figure 5B). Furthermore, flow cytometry analysis of the cell cycle distribution demonstrated that the proportion of cells entering S phase was partially restored by Dia-Exos^AntagomiR-15a-3p^ treatment compared with Dia-Exos treatment (Figure 5C and 5D). Moreover, qRT-PCR analysis indicated that *Cyclin D1* and *Cyclin D3* levels were recovered in HUVECs treated with Dia-Exos^AntagomiR-15a-3p^ (Figure 5E), as were *Bcl-2* and *Bax* levels (Figure 5F). A Transwell migration assay revealed that HUVEC migration was partly restored by Dia-Exos^AntagomiR-15a-3p^ treatment compared with Dia-Exos treatment (Figure 5G and 5H). Angiogenesis was assessed through a tube formation assay, and was found to be significantly induced following Dia-Exos^AntagomiR-15a-3p^ treatment compared with Dia-Exos treatment (Figure 5I–5K).

### MiR-15a-3p inhibition partly induced the wound healing ability of Dia-Exos *in vivo*

**Figure 6:**
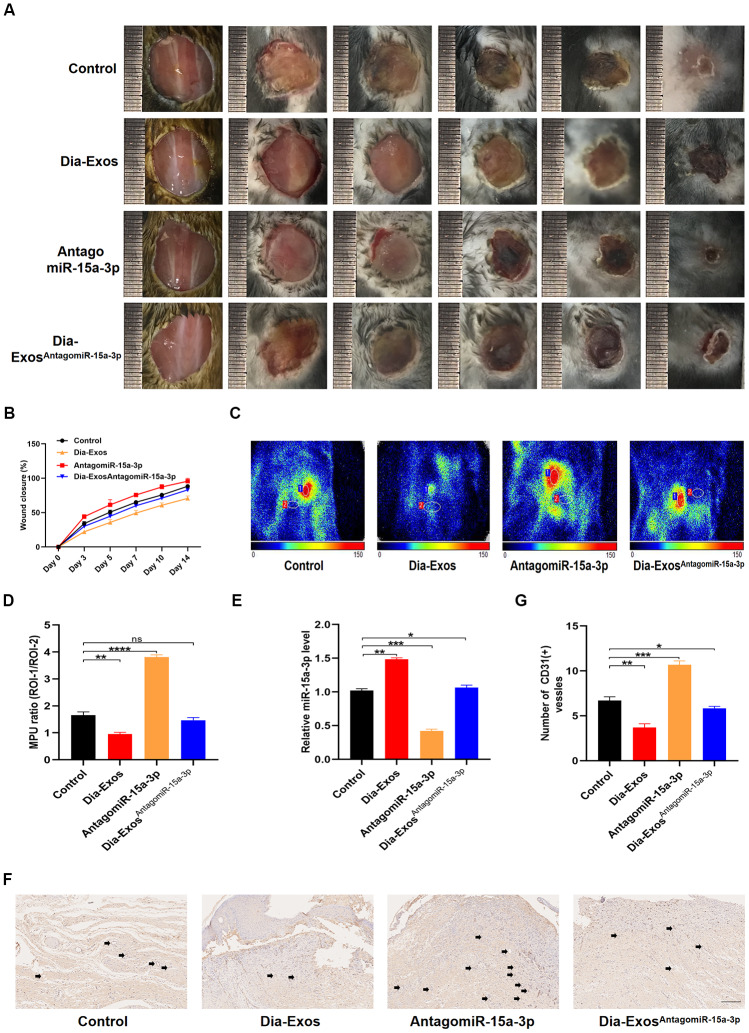
**Knockdown of miR-15a-3p partially reversed the negative effects of Dia-Exos on wound healing *in vivo.*** (**A**) General view of the wounds among in the four groups on days 0, 3, 5, 7, 10 and 14 post-wounding; n = 6 per group. (**B**) The mice of wound closure in the four groups were quantified using digital images evaluated with ImageJ software; n = 6 per group. (**C**, **D**) The blood flow at the wounds in the four groups was evaluated using small animal doppler detection; n = 6 per group. (**E**) The expression of miR-15a-3p in the wound tissues of the four groups. (**F**, **G**) CD31 immunohistochemistry results of the four groups. The number of CD31-positive cells was quantified in five random fields of view. Vessels with diameters of 2-10 μm were counted as individual vessels. n = 6 per group; scale bar: 100 μm. Data are the means ± SDs of three independent experiments. *p < 0.05, **p < 0.01, ***p < 0.001.

Next, we assessed the effects of miR-15a-3p inhibition on wound repair *in vivo*. We generated full-thickness cutaneous wounds on the backs of mice, and locally injected them with equal volumes of either PBS (Control group), Dia-Exos, antagomiR-15a-3p or Dia-Exos^AntagomiR-15a-3p^. The general appearance of the wounds indicated that wound closure was significantly better in the Dia-Exos^AntagomiR-15a-3p^ group than in the Dia-Exos group (Figure 6A and 6B). Small animal doppler detection demonstrated that the blood perfusion of the wound area was significantly greater in the Dia-Exos^AntagomiR-15a-3p^ group than in the Dia-Exos group (Figure 6C and 6D). The level of miR-15a-3p in the skin tissues was measured via qRT-PCR, and tended to be lower in the Dia-Exos^AntagomiR-15a-3p^ group than in the Dia-Exos group (Figure 6E). In addition, immunohistochemical analysis of CD31 indicated that angiogenesis was induced in the Dia-Exos^AntagomiR-15a-3p^ group compared with the Dia-Exos group (Figure 6F and 6G). All these results suggested that miR-15a-3p inhibition partly improved the wound healing ability of Dia-Exos.

### MiR-15a-3p impaired HUVEC functionality by suppressing the *NOX5/*ROS signaling pathway

**Figure 7:**
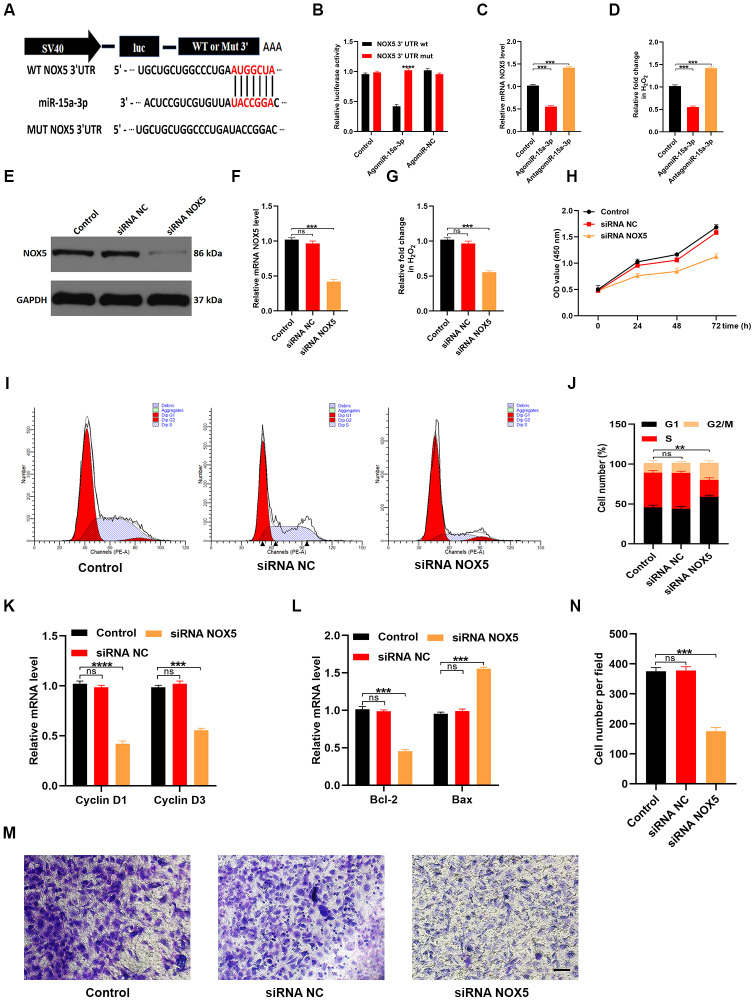
**MiR-15a-3p inhibits the *NOX5*/ROS signaling pathway.** (**A**, **B**) The binding between miR-15a-3p and *NOX5* was demonstrated with a luciferase reporter assay. (**C**) The effects of miR-15a-3p on *NOX5* levels were assessed using qRT-PCR. (**D**) The intracellular release of ROS was measured in the three groups. (**E**, **F**) WB and qRT-PCR analyses were used to detect the efficacy of *NOX5* siRNA. (**G**) The release of ROS decreased when *NOX5* was silenced. (**H**) A CCK-8 assay was applied to assess cell proliferation after different treatments. (**I**, **J**) Flow cytometry was used to quantify the cell cycle distribution in treated cells. (**K**) The proliferation-related genes *Cyclin D1* and *Cyclin D3* were assessed using qRT-PCR. (**L**) The apoptosis-related genes *Bcl-2* and *Bax* were assessed using qRT-PCR. (**M**, **N**) A Transwell migration assay was used to assess the effects of miR-15a-3p on HUVEC migration; scale bar: 100 μm. Data are the means ± SDs of three independent experiments. *p < 0.05, **p < 0.01, ***p < 0.001.

We next explored the mechanisms by which miR-15a-3p impaired HUVEC functionality. TargetScan (http://www.targetscan.org/vert_70/) was used to predict potential target genes of miR-15a-3p. Previous studies have indicated that NOX enzyme activation and ROS release are associated with cutaneous wound repair [15, 16]. After carefully screening the candidate genes, we found that *NOX5* may have been involved in the suppression of wound repair by miR-15a-3p. A luciferase assay suggested that miR-15a-3p could specifically bind to the predicted target region of *NOX5* mRNA. Accordingly, when this target region was mutated, the miRNA could no longer reduce luciferase activity (Figure 7A and 7B). *NOX5* expression was clearly suppressed and ROS release was obviously reduced when miR-15a-3p was upregulated (Figure 7C and 7D).

Next, we transfected HUVECs with siRNA specific for *NOX5* to assess whether HUVEC functionality depended on NOX5. Both *NOX5* expression and ROS release were significantly reduced in *NOX5* siRNA-treated cells (Figure 7E–7G). A CCK-8 cell proliferation assay demonstrated that *NOX5* siRNA inhibited HUVEC proliferation (Figure 7H). Flow cytometry analysis of the cell cycle distribution indicated that the proportion of cells entering S phase was significantly reduced following *NOX5* siRNA treatment (Figure 7I and 7J). Similarly, qRT-PCR analysis revealed that *Cyclin D1* and *Cyclin D3* levels were reduced in HUVECs treated with *NOX5* siRNA (Figure 7K). Consistently, *Bcl-2* levels were reduced and *Bax* levels were elevated following *NOX5* siRNA treatment (Figure 7L). A Transwell migration assay demonstrated that *NOX5* siRNA treatment impaired the migration ability of HUVECs (Figure 7M and 7N). These results suggested that miR-15a-3p inhibited wound repair by downregulating the *NOX5/*ROS signaling pathway.

## DISCUSSION

Exosomes have attracted increasing interest as nanomaterials with the potential to regulate biological processes [15–17]. Exosomes are ideal drug carriers for many diseases, due to their ability to protect their contents (e.g., nucleic acids, non-coding RNAs and proteins) and deliver them to target cells. They are also vital for paracrine and endocrine communication between different cells and organs [18–19]. A recent study described a novel biomaterial consisting of BAY55-9837-loaded exosomes coupled with superparamagnetic iron oxide nanoparticles under an external magnetic force, which seemed to be a promising peptide drug carrier for the treatment of type 2 diabetes [20]. Exosomes are abundant in circulating blood, and vascular endothelial cells (which are crucial for wound healing) can internalize circulating exosomes [21]. Therefore, exosome function is important for successful wound repair.

In this study, we investigated the influence of Dia-Exos on HUVECs *in vitro*. We found that Dia-Exos disrupted angiogenesis by impairing HUVEC proliferation, migration and tube formation, as well as inducing HUVEC apoptosis. To study the sustained influence of these exosomes *in vivo*, we applied different exosomes (Con-Exos and Dia-Exos) to the wound sites of mice every two days and observed the wound healing progression. Dia-Exos significantly reduced the number of newly generated blood vessels and the MPU ratio at the wound site. Our results demonstrated that Dia-Exos impaired HUVEC functionality, which may explain why DFU tends to persist once it has developed.

Analyses of differentially expressed miRNAs have revealed the involvement of miRNAs in DFU progression [23]. Using miRNA microarray expression data from GSE80178, we found that miR-15a-3p levels in foot skin were higher in DFU patients than in non-diabetic controls. A qRT-PCR analysis demonstrated that miR-15a-3p was also overexpressed in Dia-Exos. Recently, exosomes have been shown to secrete miRNAs, allowing their transfer to proximal or distal target cells, where they can inhibit gene expression and alter cellular functions [22, 24]. Consistent with this concept, our data suggested that the upregulation of miR-15a-3p in Dia-Exos impaired cellular function and therefore delayed wound healing. To confirm that the upregulation of miR-15a-3p in Dia-Exos was responsible for their impairment of wound healing, we used an miR-15a-3p antagonist. When miR-15a-3p was knocked down, the negative effects of Dia-Exos were partly reversed *in vitro* and *in vivo*. Therefore, we regard exosomal miR-15a-3p as a vital contributor to the inhibitory effects of Dia-Exos on wound healing.

Although this study revealed the function of exosomal miR-15a-3p, research is still needed to determine the origin of circulating exosomal miR-15a-3p. Exosomal miR-15a-3p derived from the pancreas has been reported to worsen the complications of diabetes [11], and pancreatic islet dysfunction is known to contribute to the development of diabetes. Therefore, we speculate that the pancreatic islets could be a source of exosomal miR-15a-3p.

ROS are associated with angiogenesis and cutaneous wound repair. At low levels, ROS function as signaling molecules that regulate cell growth, migration, differentiation and gene expression [25, 26]. NOX has been reported to activate redox signaling pathways that promote angiogenic responses *in vitro* and *in vivo* [27–30]. In this study, we identified *NOX5* as a potential target gene of miR-15a-3p, and verified their binding in luciferase assays. We also found that the downregulation of *NOX5* impaired the release of ROS and the functionality of HUVECs. Thus, miR-15a-3p seems to exert its effects by inhibiting the *NOX5*/ROS signaling pathway.

**Figure 8:**
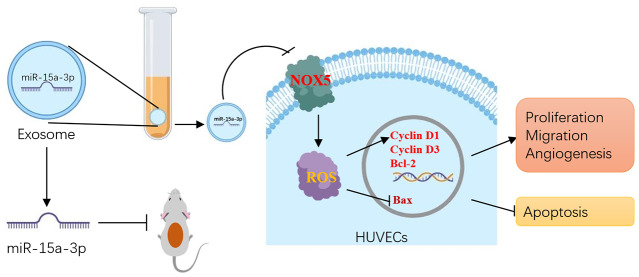
**Schematic diagram of the proposed mechanisms by which inhibiting circulating exosomal miR-15a-3p enhanced the angiogenesis and survival of HUVECs.**

In summary, this study demonstrated that Dia-Exos impaired wound healing due to their enrichment of miR-15a-3p, which suppressed the *NOX5*/ROS signaling pathway (Figure 8). Our findings suggest that the application of nanomaterials combined with miR-15a-3p-inhibitors may be a feasible and promising therapeutic strategy to accelerate DFU healing in the future.

## MATERIALS AND METHODS

### Ethical statement

Informed consent was obtained before this study. The study protocols were approved by the Ethics Committee of Wuhan Union Hospital, Tongji Medical College, Huazhong University of Science and Technology. All experimental animal protocols were performed in compliance with the Guide for the Care and Use of Laboratory Animals, and were approved by the Animal Care Committee of Wuhan Union Hospital, Tongji Medical College, Huazhong University of Science and Technology.

### Animal wound model and administration

To eliminate gender effects, we purchased male C57BL/6J mice (six weeks old, weighing 20-30 g) from The Center of Experimental Animals, Tongji Medical College, Huazhong University of Science and Technology. Intraperitoneal pentobarbital sodium (50 mg/kg; Sigma-Aldrich, MO, USA) was used to anesthetize the animals. The mice were shaved, and a full-thickness excisional skin wound (10 mm in diameter) was produced on the upper back of each mouse. Then, the mice were randomly divided into five groups: 1) Control group (wounds treated with 100 μL PBS); 2) Con-Exos group (wounds treated with 200 μg Con-Exos in 100 μL PBS); 3) Dia-Exos group (wounds treated with 200 μg Dia-Exos in 100 μL PBS); 4) AntagomiR-15a-3p group (wounds treated with 2 OD antagomiR-15a-3p in 100 μL diethyl pyrocarbonate-treated water); and 5) Dia-Exos^antagomiR-15a-3p^ group (wounds treated with 2 OD antagomiR-15a-3p in 100 μL diethyl pyrocarbonate-treated water and 200 μg Dia-Exos in 100 μL PBS). Briefly, the mice were subcutaneously injected around the wounds at four injection sites (25 μL per site) on days 0, 3, 5, 7, 9 and 11 post-wounding (n = 6). On days 0, 3, 5, 7, 10 and 14 post-wounding, the wounds were photographed and measured with a caliper rule.

### Measurement of wound closure rate and newly formed blood vessels

The wound closure rate was assessed on days 0, 3, 5, 7, 10 and 14. Digital images of the wounds were evaluated with ImageJ software (National Institutes of Health). On day 14 post-injury, skin specimens were collected, fixed with 4% paraformaldehyde, dehydrated with graded ethanol, embedded in paraffin and then cut into 10-μm-thick sections.

For CD31 immunohistochemistry, antigen retrieval was performed for 15 min, and then the samples were blocked for 30 min with goat serum. Subsequently, an anti-CD31 antibody (1:100; Abcam, UK) was applied to the samples overnight at 4 °C. The number of CD31-positive cells was counted in five random fields, and vessels with diameters of 2-10 μm were counted as individual newly generated vessels.

### Small animal doppler detection

On day 10 post-operation, a laser speckle contrast imaging system was used to evaluate blood perfusion. Briefly, a PeriCam PSI-ZR system (PERIMED Ltd, Stockholm, Sweden) was used to obtain images of each wound. The perfusion units were determined with an invisible near-infrared laser (785 nm) for blood perfusion measurements. Images were captured using the same scan area dimensions at a constant distance from the wound surface. Flux images of each wound site were analyzed with PIMSoft (Moor Instruments Ltd, Axminster, UK), and the MPU ratio was calculated as the MPU of the wound area (region of interest 1) divided by the MPU of the area around the wound (region of interest 2).

### Cell culture and transfection

HUVECs were purchased from the Cell Bank of the Chinese Academy of Sciences (Shanghai, China), and were cultured in RPMI 1640 medium (Thermo Fisher Scientific, MA, USA) containing 10% exosome-depleted fetal bovine serum (FBS; BI Israel). Lipofectamine 3000 (Thermo Fisher Scientific) was used according to the provided directions for cell transfection. Cells were cultured at 37 °C with 5% CO_2_ and 95% humidity. For the agomir and antagomir transfection steps, constructs were obtained from GenePharma (Shanghai) and used at a concentration of 200 mM. The same approach was used for the miRNA and siRNA oligo transfections, except that the constructs were used at 50 nM.

### CCK-8 assay

HUVECs (5×10^3^) were added to 96-well plates and cultured for 24, 48 or 72 h. Then, CCK-8 reagent (#96992, Sigma-Aldrich) was added to the cells in serum-free medium. The cells were incubated for 2 h, and the absorbance was measured at 450 nm.

### Cell scratch assay

HUVECs (2×10^5^ cells per well) were seeded into a 12-well plate and incubated at 37 °C. After the cells had attached, the monolayer was scratched with a 200-μL pipette tip, washed with PBS to remove floating cells, and then exposed to different treatments. The cells were photographed at 0, 24, 36 and 48 h post-scratch. The migration rate was calculated as the ratio of the closure area to the initial wound area.

### Transwell migration assay

For the Transwell migration assay, 24-well Transwell inserts (#140629, Thermo Fisher Scientific) were used with 8-μm-pore-sized filters. HUVECs (1×10^4^ cells per well) were suspended in low-serum medium (containing 5% FBS) and plated into the upper chamber. The lower chamber was filled with 500 μL of complete medium (containing 10% FBS) supplemented with different treatments. After 12 h of incubation, the cells attached to the upper surface of the filter membrane were removed with cotton swabs. The cells on the bottom side of the filter (the migrated cells) were stained with 0.5% crystal violet for several minutes and quantified under an optical microscope.

### Tube formation assay

For the tube formation assay, 60 μL of cold Matrigel (#354234, Corning, NY, USA) was transferred to each well of a 96-well plate and incubated at 37 °C for 40 min. HUVECs (2×10^4^ per well) were then added to the Matrigel-coated plate and randomly assigned to different treatment groups. The cells were cultured for 8 h, and then three random fields of view were captured with an inverted microscope. The tube length and total branch points were quantified with ImageJ software.

### Luciferase reporter assay

The portion of the 3’ untranslated region (UTR) of *NOX5* mRNA containing the putative target site of miR-15a-3p (nucleotides 4633-4640) was determined using TargetScan (version 7.0; http://www.targetscan.org/vert_70/). Then, PCR was used to amplify this target site in cDNA from HUVECs, and the sequence was ligated into the pGL3-basic vector (Promega Corporation). To create the pGL3-*NOX5*-3’UTR-mutant (Mut) vector, we introduced two site mutations into the potential miR-15a-3p target site using a QuikChange Site-Directed Mutagenesis kit (Agilent Technologies, Inc.).

Next, Lipofectamine® 3000 (Thermo Fisher Scientific) was used to co-infect HUVECs with the *Renilla* plasmid and either pGL3-*NOX5*-3’UTR-wild-type (200 ng) or pGL3-*NOX5*-3’UTR-Mut (200 ng). The cells were then transfected with the miRNA negative control mimic (10 nM) or miR-15a-3p mimic (10 nM) for 48 h at 37 °C. The miRNA negative control mimic and miR-15a-3p mimic transfection kits were supplied by Shanghai GenePharma Co., Ltd. A Dual-Luciferase Reporter assay system (Promega Corporation) was used to measure the relative luciferase activity of each well. Firefly luciferase expression was normalized to that of *Renilla*.

### qRT-PCR analysis

TRIzol® reagent (Thermo Fisher Scientific) was used to isolate total RNA from cell and tissue samples. Then, ReverTra Ace® qPCR RT Master Mix (Toyobo Life Science) was used to reverse-transcribe the purified RNA into cDNA, in accordance with the manufacturer’s protocol. Reverse transcription was conducted for 15 min at 42 °C and 5 min at 98 °C, and the reaction volume was 20 μL. The qRT-PCR thermocycling conditions included an initial denaturation at 95 °C for 30 sec, followed by 40 cycles of 95 °C for 5 sec and 60 °C for 30 sec, and the reaction volume was 25 μL. The relative miRNA levels were normalized to *GAPDH* levels (the internal control) and were calculated according to the 2^-ΔΔCt^ method. All experiments were conducted in triplicate.

The primer sequences were: miR-15a-3p, forward, CTGCAGGCCATATTG TGCTGCCTCA, reverse, GTGCAGGGTCCGAGGT; U6, forward, CTCGCTTCGGCAGCACA, reverse, AACGCTTCACGAATTTGCGT; *Bcl-2*, forward, GATAACGGAGGCTGGGATGC, reverse, TCACTTGTGGCCCAGATAGG; *Bax*, forward, CCCTTTTGCTTCAGGGTTTC, reverse, GAGACACTCGCTCAGCTTCTTG; *Cyclin D1*, forward, TTGCCCTCTGTGCCACAGAT, reverse, TCAGGTTCAGGCCTTGCACT; *Cyclin D3*, forward, CTGGCCATGAACTACCTGGA, reverse, CCAGCAAATCATGTGCAATC; *NOX5*, forward, AGTATCATGTACAGGCACCA, reverse, GTTGTCTTGGACACCTTCG; *GAPDH*, forward, CCGTTGAATTTGCCGTGA, reverse, TGATGACCCTTTTGGCTCCC.

### WB analysis

The cells were washed three times with PBS, and radioimmunoprecipitation assay lysis buffer (Aspen Pharmacare Holdings Ltd.; cat. no. AS1004) was used to extract the total proteins from the cells. The cell lysates (1x10^4^) were subjected to 10% sodium dodecyl sulfate polyacrylamide gel electrophoresis, and the protein concentration was determined through the bicinchoninic acid method. The proteins (50 μg) were then transferred onto a 10% sodium dodecyl sulfate polyvinylidene difluoride membrane. The membrane was blocked with 5% bovine serum albumin (Abcam) at room temperature for 2 h. A chemiluminescence detection system (Canon, Inc.; cat. no. LiDE110) was then used to visualize the proteins based on the provided instructions. The following antibodies were used: anti-TSG101 (1:1000; Abcam, cat. no. Ab125011), anti-CD9 (1:1000; Abcam, cat. no. Ab92726), anti-NOX5 (OCN; 1:500; Abcam, cat. no. Ab198213) and anti-GAPDH (1:10,000; Abcam, cat no. Ab37168). All experiments were conducted in triplicate.

### Cellular ROS generation

The ROS-Glo^TM^ H_2_O_2_ assay (Promega) was used to assess cellular H_2_O_2_ levels according to the manufacturer’s instructions. The plate was read using a Glo-Max luminometer with the CellTiter-Glo built-in protocol (PMT activated). Menadione (20 μM) was used as a positive experimental control.

### Flow cytometry assay

Cell cycle progression was determined via propidium iodide staining. Cells were stained based on the provided directions and assessed via flow cytometry.

### Exosome isolation and characterization

Peripheral blood was collected from non-diabetic foot wound patients and DFU patients aged 45-60 years. To eliminate the potential effects of gender, we only used peripheral blood from men. Blood was isolated in tubes containing citrate phosphate dextrose and spun at 3000×*g* for 15 min. The serum was collected and spun again under the same conditions to remove any remaining platelets. The supernatants were centrifuged at 10,000×*g* for 30 min and then ultracentrifuged at 100,000×*g* for 70 min. The exosome pellets were washed three times with PBS and ultracentrifuged under the same conditions, after which the pellets were resuspended in 15 mL of PBS and filtered through a 0.2-μm filter (122-0020PK, Thermo Fisher Scientific). The samples were then subjected to ultrafiltration through a 15-mL Amicon Ultra-15 Centrifugal Filter (Millipore, MA, USA) in a centrifuge at 4000×*g* to a final volume of 200 μL.

The purified exosomes were combined with 4% osmium tetroxide to a total volume of 50 μL and left for 30 min at 4 °C, after which they were added to a copper grid and stained with 1% phosphotungstic acid. TEM (EFI, TECNAI G2) was then used to assess their morphology. A Nanosizer™ instrument (Malvern Instruments, Malvern, UK) was used for DLS analyses, and WB was used to assess exosomal surface marker expression. The miR-15a-3p levels in the exosomes were analyzed through qRT-PCR. For the aforementioned experiments, we analyzed the exosomes isolated from each subject (n = 10, diabetic vs. normal) individually. For further cell and animal experiments, we pooled the exosomes for each group (concentration: 50 μg/mL). Specifically, the effects of Dia-Exos on HUVECs were verified in three independent experiments using the combined exosomes. The effects of Dia-Exos on *in vivo* wound healing were also evaluated using the combined exosomes.

### Exosome uptake assay

The red dye PKH26 (Sigma-Aldrich) was used to stain membranes and track purified exosomes. Then, the exosomes were washed with 20 mL of PBS and recollected via ultracentrifugation. The exosomes were combined with HUVECs, and immunofluorescence used to measure the cellular uptake of the labeled particles.

### Microarray data from patients with diabetes

Microarray data from three diabetic patients and three normal controls were retrieved from a previously performed NanoString nCounter microRNA expression assay (NanoString Technologies, Seattle, WA, USA) publicly deposited in the NCBI GEO (http://www.ncbi.nlm.nih.gov/geo) under the accession number GSE80178.

### Differentially expressed miRNAs

The raw miRNA expression data were imported into R software (version 3.6.1), and were log2 transformed and quantile normalized. Differential expression was analyzed with GEO2R (https://www.ncbi.nlm.nih.gov/geo/geo2r/). P values < 0.05 and log2 fold-change values > 1 were considered statistically significant for differentially expressed miRNAs.

### Statistical analysis

The data are presented as the mean ± standard deviation (SD). Student’s t-test was applied to compare two groups, while one-way analysis of variance with Tukey’s post hoc test was used to compare more than two groups. Statistical analyses were conducted with GraphPad Prism 8.0. P < 0.05 was the significance threshold.

### Ethical approval

The Ethics Committee of Union Hospital, Tongji Medical College, Huazhong University of Science and Technology approved of this study.

## Supplementary Material

Supplementary Figure 1
