# Correction for: Inhibition of circulating exosomal microRNA-15a-3p accelerates diabetic wound repair

**DOI:** 10.18632/aging.205514

**Published:** 2024-04-15

**Authors:** Yuan Xiong, Lang Chen, Tao Yu, Chenchen Yan, Wu Zhou, Faqi Cao, Xiaomeng You, Yingqi Zhang, Yun Sun, Jing Liu, Hang Xue, Yiqiang Hu, Dong Chen, Bobin Mi, Guohui Liu

**Affiliations:** 1 Department of Orthopedics, Union Hospital, Tongji Medical College, Huazhong University of Science and Technology, Wuhan 430022, China; 2 Department of Orthopedic Surgery, Tongji Hospital, Tongji University School of Medicine, Shanghai 200065, China; 3 Department of Orthopedic Surgery, Brigham and Women’s Hospital, Harvard Medical School, Boston, MA 02125, USA; 4 Department of Neurosurgery, Union Hospital, Tongji Medical College, Huazhong University of Science and Technology, Wuhan 430022, China

**Keywords:** microRNA-15a-3p, exosome, diabetic foot ulcer, wound repair, NADPH oxidase 5

**This article has been corrected:** The authors found an error in **Figure 5G**, which depicts transwell migration assays assessing the effects of miR-15a-3p inhibition on HUVEC cells. The central panel, illustrating migration of HUVECs treated with exosomes derived from DFU individuals (Dia-Exos), was replaced with the appropriate image from the original set of experiments. This inadvertent error does not in any way compromise the validity or integrity of the study’s findings. The authors take full responsibility for this oversight and sincerely apologize for any inconvenience caused.

The corrected **Figure 5** is presented below.

**Figure 5:**
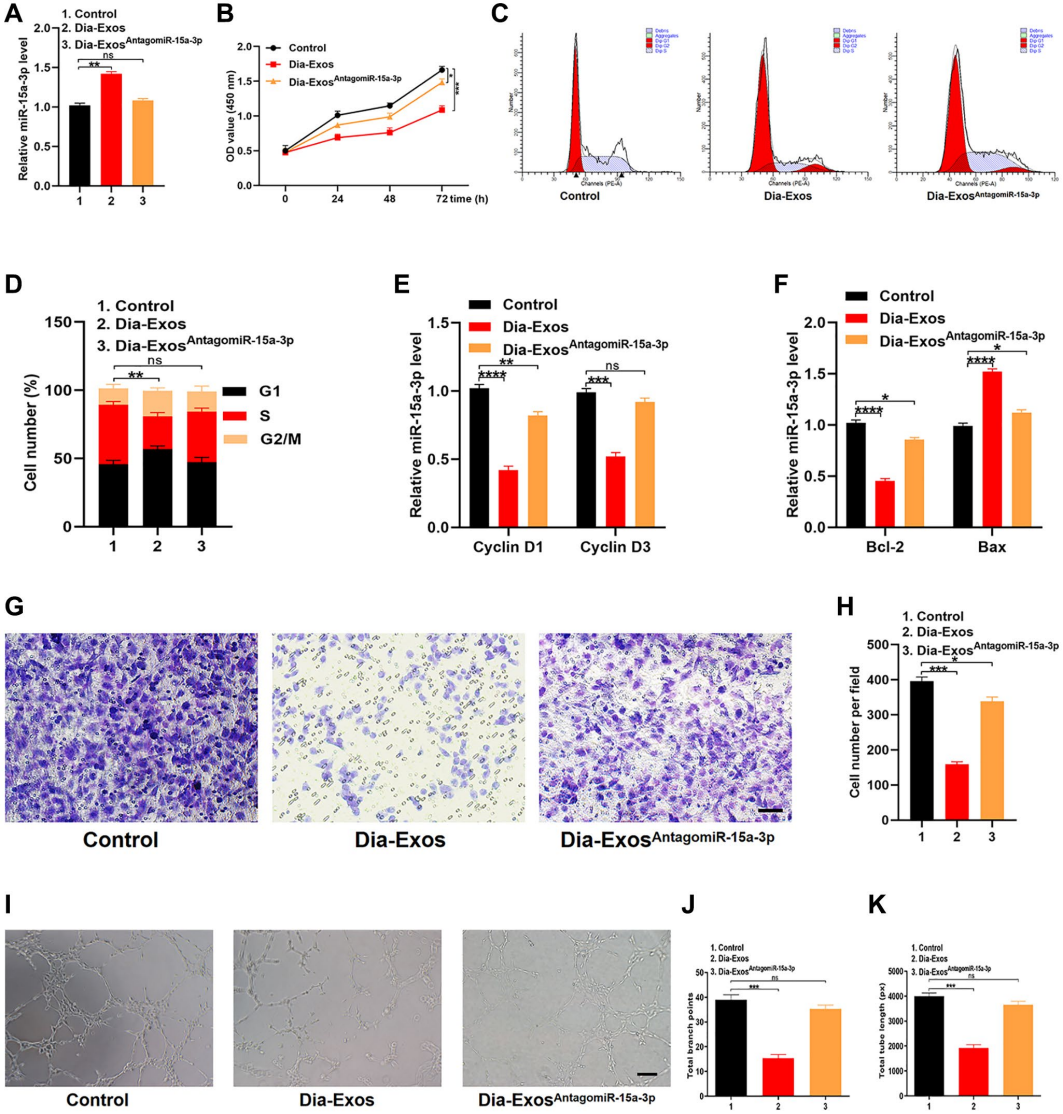
**Inhibition of miR-15a-3p partially reversed the impaired functionality of HUVECs treated with Dia-Exos.** (**A**) MiR-15a-3p levels in the three groups were measured using qRT-PCR. (**B**) CCK-8 assay results of the three groups. (**C**, **D**) Flow cytometry was used to quantify the cell cycle distribution in treated cells. (**E**) The qRT-PCR results of the proliferation-related genes *Cyclin D1* and *Cyclin D3*. (**F**) The apoptosis-related genes *Bcl-2* and *Bax* were assessed using qRT-PCR. (**G**, **H**) A Transwell migration assay was used to assess the effects of miR-15a-3p inhibition on HUVEC migration; scale bar: 100 μm. (**I**–**K**) A tube formation assay was used to assess the effects of miR-15a-3p inhibition on HUVEC angiogenesis; scale bar: 200 μm. Data are the means ± SDs of three independent experiments. ^*^p < 0.05, ^**^p < 0.01, ^***^p < 0.001.

