# Supplementary material for: Inhibition of circulating exosomal microRNA-15a-3p accelerates diabetic wound repair
Source: Aging (Albany NY). 2020 May 21;12(10):8968–86. doi: 10.18632/aging.103143 (PMC7288917; doi:10.18632/aging.103143)
Supplement: Supplementary Figure 1 [file aging-12-103143-s001..pdf]

## SUPPLEMENTARY FIGURE

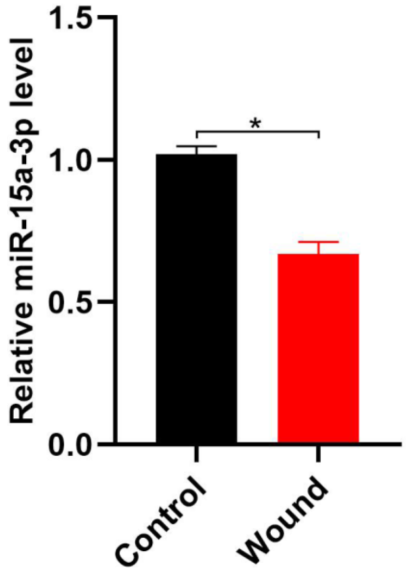


**Supplementary Figure 1. The expression level of miR-15a-3p in HUVECs with different treatments.** The scratch HUVECs injury was induced in a 24-well Petri dishes, which were manually scratched with a 10 µl plastic stylet needle following a 4×4 square grim (with 4-mm space between each line). This scratch injury was regarded as wound group, and untreated cells were used as controls.
